# Dynamic transcriptome landscape in the song nucleus HVC between juvenile and adult zebra finches

**DOI:** 10.1002/ggn2.10035

**Published:** 2021-01-06

**Authors:** Zhimin Shi, Zeyu Zhang, Lana Schaffer, Zhi Huang, Lijuan Fu, Steven Head, Terry Gaasterland, Xiu‐Jie Wang, XiaoChing Li

**Affiliations:** ^1^ Neuroscience Center of Excellence Louisiana State University School of Medicine New Orleans Louisiana USA; ^2^ Key Laboratory of Genetic Network Biology Institute of Genetics and Developmental Biology, Chinese Academy of Sciences Beijing China; ^3^ University of Chinese Academy of Sciences Beijing China; ^4^ Scripps Research Institute La Jolla California USA; ^5^ University of California at San Diego La Jolla California USA; ^6^ Present address: California Medical Innovations Institute San Diego California USA

**Keywords:** circuit development, gene expression, HVC, RNA‐Seq, vocal communication, zebra finch

## Abstract

Male juvenile zebra finches learn to sing by imitating songs of adult males early in life. The development of the song control circuit and song learning and maturation are highly intertwined processes, involving gene expression, neurogenesis, circuit formation, synaptic modification, and sensory‐motor learning. To better understand the genetic and genomic mechanisms underlying these events, we used RNA‐Seq to examine genome‐wide transcriptomes in the song control nucleus HVC of male juvenile (45 d) and adult (100 d) zebra finches. We report that gene groups related to axon guidance, RNA processing, lipid metabolism, and mitochondrial functions show enriched expression in juvenile HVC compared to the rest of the brain. As juveniles mature into adulthood, massive gene expression changes occur. Expression of genes related to amino acid metabolism, cell cycle, and mitochondrial function is reduced, accompanied by increased and enriched expression of genes with synaptic functions, including genes related to G‐protein signaling, neurotransmitter receptors, transport of small molecules, and potassium channels. Unexpectedly, a group of genes with immune system functions is also developmentally regulated, suggesting potential roles in the development and functions of HVC. These data will serve as a rich resource for investigations into the development and function of a neural circuit that controls vocal behavior.

## Graphical abstract

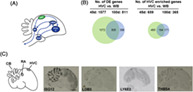

## 1 INTRODUCTION

Zebra finches (*Taeniopygia guttata*) use songs and calls to communicate with members of their species. Male juvenile zebra finches learn to sing during a developmentally restricted period early in life by imitating songs of conspecific adult males. Typically, juveniles begin to vocalize at around 30 days of age (30 d). Initially, their songs are highly variable. Through a sensory‐motor learning process that spans about 2 months, juveniles hear an adult tutor's song, practice singing, and use auditory feedback to match their own vocal output with the tutor song. By 90 days of age, male zebra finches reach sexual maturity,^1^ and their song matures into a stereotyped adult song. This newly acquired adult song, although displaying some variability, resembles the tutor song, and the bird sings this adult song throughout life.^2^, ^3^, ^4^, ^5^, ^6^

**FIGURE 1:**
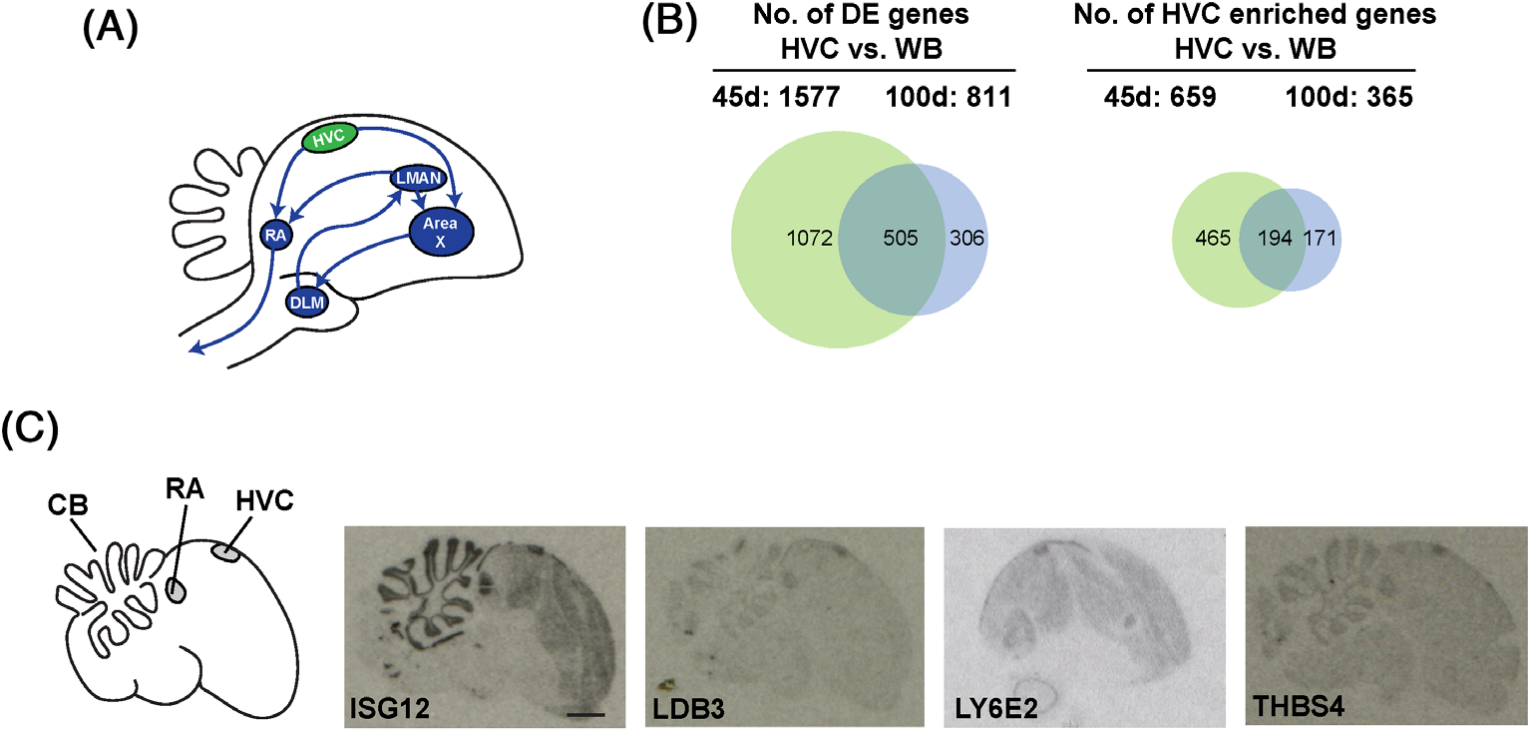
Comparison of gene expression in the HVC and whole brain tissue. A, A schematic diagram showing the song control circuit with major song nuclei and the connections among them. This study focused on the HVC, highlighted in green. B, Left, Venn diagram showing the number of genes differentially expressed in the HVC relative to the whole brain tissue at 45 d and 100 d. Right, Venn diagram showing the number of genes with enriched expression in the HVC relative to the whole brain tissue at 45 d and 100 d (*q* < 0.1). DE, differentially expressed. WB, whole brain. C, Images of in situ hybridization on sagittal brain sections showing enriched expression of *ISG12*, *LBD3*, *LY6E2*, and *THBS4* in the HVC of 45 d male zebra finches. Scale bar, 1.5 mm

Song behavior is controlled by a group of interconnected brain nuclei commonly referred to as the song system (Figure 1A). The song system consists of two distinct pathways: the motor pathway, which controls song production, and the anterior forebrain pathway (AFP), which is necessary for song learning.^7^, ^8^, ^9^, ^10^ HVC is a cortical nucleus at the junction of these two pathways.^11^, ^12^, ^13^ Neurophysiological, lesion, and imaging studies suggest that HVC encodes the spectral features and temporal patterns of a song.^10^, ^14^, ^15^, ^16^, ^17^ It is also known that before 45 days of age, when juveniles sing a poorly structured subsong, HVC is not required for singing, and singing is controlled by the anterior forebrain nucleus lMAN. After 45 d, song control gradually transfers from lMAN to HVC.^16^ The process of shifting functional dominance is accompanied by structural development in HVC. HVC begins to establish functional synapses with its downstream nucleus RA at about 35 d,^18^ and the volume of HVC and the number of neurons in HVC continue to increase^19^, ^20^ in parallel with changes in electrophysiological firing properties of HVC neurons.^5^

In recent years, applying molecular and genomics tools such as in situ hybridization and cDNA microarrays to birdsong research, progress has been made in understanding gene expression programs relevant to gender, age, brain regions, learning experiences, and song behavior.^21^, ^22^, ^23^, ^24^, ^25^, ^26^, ^27^, ^28^, ^29^, ^30^ However, the dynamic transcriptional landscapes in HVC that enable its structural and functional changes as juveniles mature to adulthood remain enigmatic. In this study, we profiled transcriptomes in the HVC of male zebra finches using RNA‐Seq; we focused on two age groups: 45 d, when motor control of song begins to transfer to HVC, and juveniles sing a highly variable song; and 100 d, when song learning is complete, and the now adult zebra finches sing a mature adult song.^3^, ^4^, ^16^ Using a combination of bioinformatics analysis and experimental validation, we identified gene repertoires that define molecular signatures of HVC at 45 d and 100 d. We also describe the dynamic changes in gene expression that occur in HVC as zebra finches mature. These results provide unique insights into the transcriptional landscape underlying the development and functional maturation of the neural circuit for vocal communication.

## 2 RESULTS

### 2.1 Profiling transcriptomes in HVC by RNA‐Seq

To survey gene expression and transcriptome changes in HVC during song development, we collected brain tissues of 45 d and 100 d male zebra finches obtained from our breeding colony. Juveniles were reared with their parents in breeding cages until the collection time. The newly adult birds were separated from their parents at around 60 to 70 d, and subsequently kept in group cages until brain collection. It is known that moment‐to‐moment sensory‐motor experiences, such as singing or hearing songs, induce changes in gene expression in song related brain regions.^31^, ^32^ To obtain a gene expression profile in HVC at basal levels that reflects developmental stages, we monitored the birds in the morning for one hour prior to brain collection to verify that they did not sing and did not hear songs. We dissected out HVC tissue from sagittal brain sections under a dissecting microscope. We isolated total RNAs, and pooled RNA samples from HVC tissues of four animals for making each cDNA library (see the methods section for details). To identify genes with enriched expression in HVC relative to the rest of the brain, we also made cDNA libraries from whole brain tissue (WB) of male zebra finches at 45 d and 100 d. We sequenced these cDNA libraries using the Illumina GAII platform, which produced over 30 million raw sequence reads of 79 nt per library.

After filtering and trimming adaptor sequences, we obtained a total of 150 million high quality sequence reads from all libraries combined. We mapped these reads to the zebra finch genome assembly (3.2.4/taeGut1) using the Eland (Illumina) software package. Typically, we obtained 25‐30 million high quality sequence reads for each library; among them, 50% to 60% were mapped to the zebra finch genome. About 15% to 25% of the mapped reads were mapped to exons of annotated genes, while the remaining reads were mapped to intronic, intergenic regions, and/or regions without annotation. Altogether, mapped genes represent about 50% of the total number of annotated zebra finch genes. The general characteristics of library samples, sequencing, and mapping results, including the read counts of each libraries, their mapping rates to the genome, and the number of genes covered by each sequenced sample, are summarized in Table S1.

### 2.2 RNA‐Seq analysis reveals distinct gene expression patterns in juvenile and adult HVC

We first compared gene expression profiles in HVC with those in the whole brain samples, which allowed us to identify a large number of genes with enriched expression in the HVC of juvenile and adult finches. Using a cutoff of *q* < 0.1 (FDR‐adjusted *p*‐value), at 45 d, 1577 genes were differentially expressed in HVC relative to the whole‐brain sample, and at 100 d, 811 genes were differentially expressed. Among these, less than half, 659 and 365 genes, respectively, showed enriched expression in HVC in juveniles and adults (Figure 1B and Data S1 and Data S2). These HVC gene expression patterns, distinct from the average gene expression in the whole brain tissue, define the transcriptional programs in the HVC at 45 d or 100 d. The larger number of genes with enriched expression in HVC at 45 d indicates higher transcriptional activities at 45 d, and suggests that gene expression at 45 d is not synchronized with the rest of the brain. The asynchronicity and transcriptional activity in HVC are gradually reduced as birds mature to adulthood, suggesting that chronological age plays a determinative role in regulation of gene expression in HVC.

The enriched expression of many genes in adult HVC, especially those expressed at high levels (eg, *ALDH1A2*, *CCNB*, *CADPS2*, *CRHBP*, *GLAR2*, *MUSTN1*, *NTS*, *PVBL*, *RELN*, etc.), have been reported previously using various experimental platforms including differential display, cDNA microarray, and/or in situ hybridization,^24^, ^25^, ^29^, ^33^, ^34^ supporting the robustness of the present study. We focused on a few genes showing enriched expression in 45 d HVC, and performed in situ hybridization to verify their expression. Consistent with the RNA‐Seq results, in situ hybridization revealed enriched expression of *ISG12*, *LDB3*, *LY6E2*, and *THBS4* in HVC compared to other brain regions at 45 d (Figure 1C). These results further show the suitability of our dissection method for isolating HVC‐specific tissue. The in situ hybridization results also revealed gene expression patterns in areas in addition to HVC. For example, on a sagittal brain section, *ISG12*, *LDB3*, and *THBS4* also showed enriched expression in RA, a song nucleus acting downstream from HVC, which, together with HVC, controls song related motor activities. These expression patterns suggest that gene expression in the song control circuit might be functionally segregated. In an analysis of cDNA microarray gene expression data in several song‐related brain regions, Lovell et al observed that each song‐related region has a distinct gene expression pattern compared to its adjacent region, and their data also suggest that HVC and RA share a large number of co‐expressed genes.^25^ Together, these data hint at the ontogeny and evolutionary history of the song control circuit.

**FIGURE 2:**
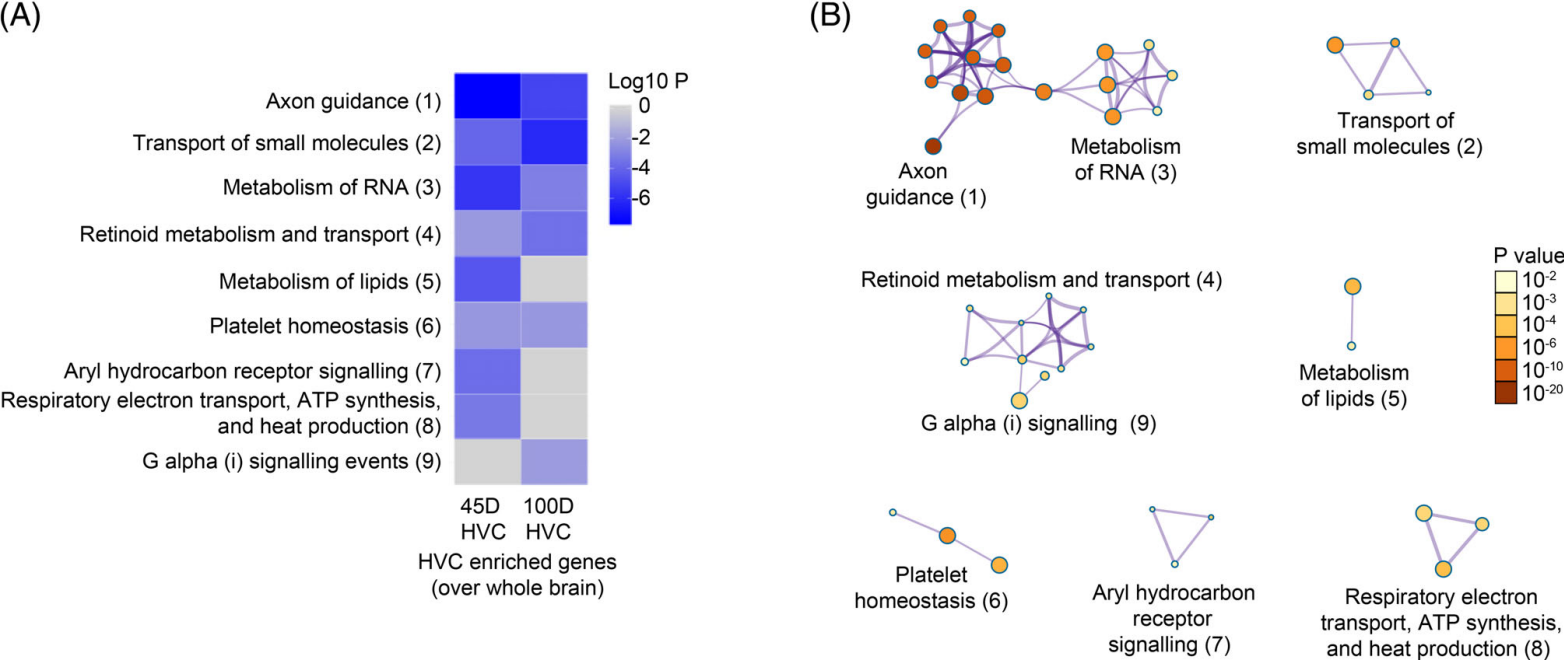
REACTOME enrichment analysis of genes in the HVC compared with whole brain at 45 d and 100 d. A, Enriched REACTOME terms among genes that show enriched expression in 45 d or 100 d HVC compared to whole brain tissues. B, Interconnections between enriched terms. In both A and B, *p*‐values are color‐coded; grey represents the absence of enrichment

To gain more insight into the biological functions of the differentially expressed genes, we performed REACTOME pathway enrichment analysis of genes with enriched expression in HVC at both 45 d and 100 d. The term axon guidance is most highly enriched in 45 d HVC and to a lesser extend in 100 d HVC. This group includes genes coding for calcium channel, potassium channel, NMDA receptors, endocytosis proteins, extracellular adhesion proteins, RELN signaling, ROBO and Slit signaling, and many ribosomal and proteasome proteins. The term metabolism of RNA is also highly enriched in 45 d HVC. This group includes many genes with functions related to mRNA splicing, processing of capped intron‐containing pre‐mRNA, cleavage of growing transcript in the termination region, and mRNA 3′‐end processing. Other terms enriched in 45 d HVC include metabolism of lipids, aryl hydrocarbon receptor signaling, respiratory electron transport, and ATP synthesis (Figure 2A). Fewer terms are significantly enriched in adult HVC than in juvenile HVC. The most highly enriched term in adult HVC is transport of small molecules. This term includes a large group of genes related to ion transporters or ion exchangers for sodium, potassium, or chloride and amino acid transporters. Other terms enriched in adult HVC are retinoid metabolism and G protein coupled signaling events (Figure 2A). Details of genes associated with each term can be found in Data S3. Interestingly, gene network analysis indicated that terms related to RNA metabolism and axon guidance were connected (Figure 2B), suggesting that posttranscriptional mRNA processing such as RNA splicing may play a role in regulating axon guidance or growth.

### 2.3 Developmentally regulated gene expression in HVC

**FIGURE 3:**
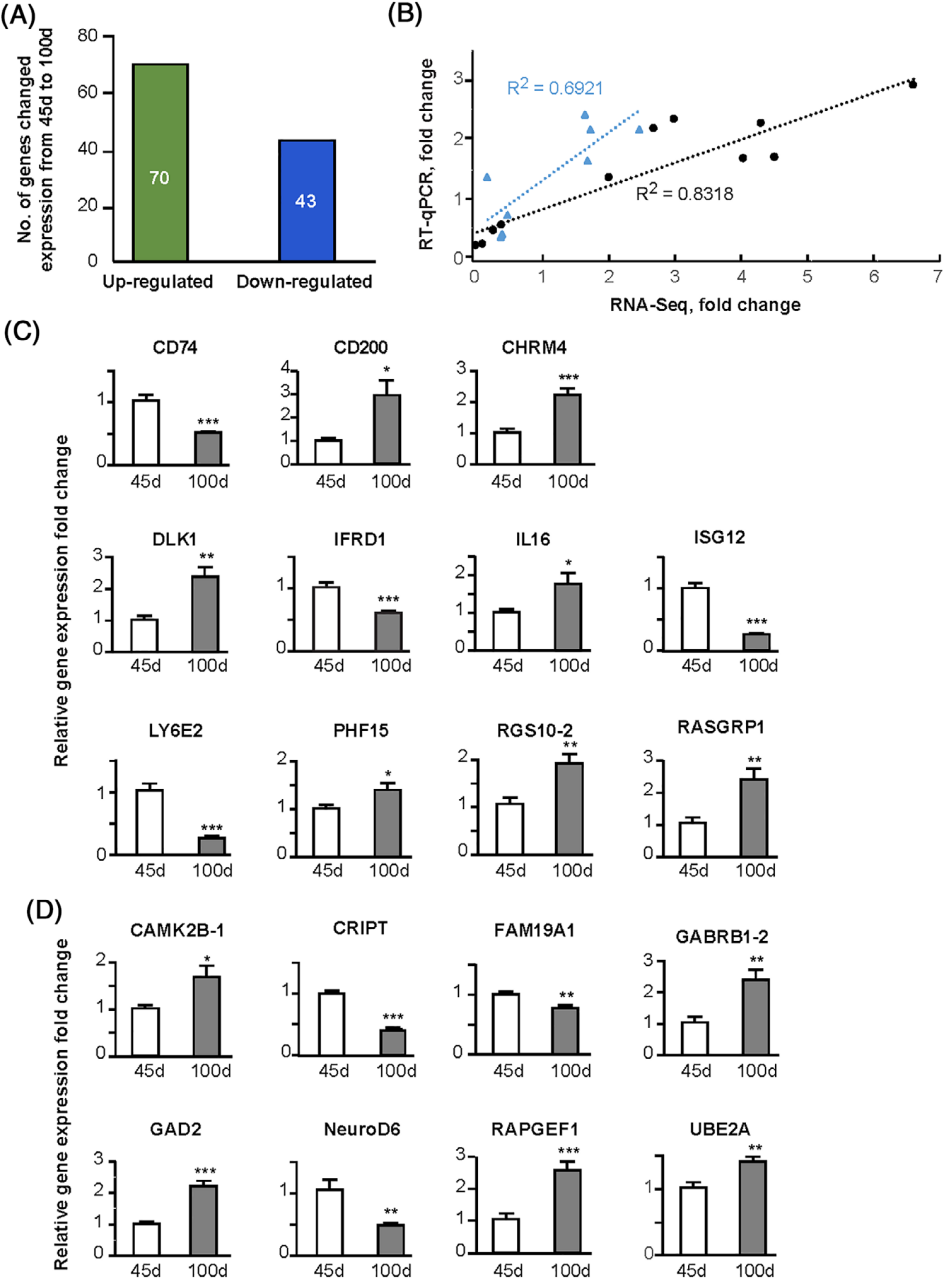
Validation of gene expression changes using RT‐qPCR. A, RNA‐Seq analysis identified 113 differentially expressed genes in HVC as birds matured from 45 d to 100 d; 70 genes increased expression, and 43 genes decreased expression (*q* < 0.1). B, Regression analysis of RNA‐Seq and RT‐qPCR results showing correlations between gene expression changes. Note the black line and the dots represent genes whose expression changes met the *q* < 0.1 cutoff in RNA‐Seq experiment. The blue line and the triangles represent genes whose expression changes did not meet the *q* < 0.1, but met the *p* < 0.01 cutoff in RNA‐Seq. C, Validation by RT‐qPCR of expression changes of specific genes that met the *q* < 0.1 cutoff in RNA‐Seq experiment (black dots in B). D, Validation by RT‐qPCR of expression changes of genes that did not meet the *q* < 0.1 cutoff, but had *p* < 0.01 in RNA‐Seq (blue triangles in B). In both C and D, n = 4‐9 animals per age group. *p < 0.05, **p < 0.01, ***p < 0.001

We examined gene expression changes in HVC between 45 d and 100 d. At a significance level of *q* < 0.1, 113 genes changed their expression between 45 d and 100 d. Among them, 70 genes increased expression and 43 genes decreased expression in HVC as juveniles matured into adulthood (Figure 3A). When we lowered the significance threshold to *p* < 0.01, 330 genes changed expression; 189 genes increased expression, and 141 genes decreased expression (Data S4 lists the genes, including their annotations, chromosomal locations, and read counts). These findings indicate that large‐scale reorganization of the transcriptome landscape occurs in HVC during the day 45 and day 100 interval when the song circuit maturation and song learning occur.

To verify the RNA‐Seq gene expression results, we performed quantitative real‐time PCR (RT‐qPCR) for a handful genes that met with the *q* < 0.1 cutoff to assess their expression levels in a separate set of HVC samples. This group of genes included *CD74*, *CD200*, *CHRM4*, *DLK1*, *IFRD1*, *IL16*, *ISG12*, *LY6E‐2*, *PHF15*, *RGS10‐2*, and *RASGRP1*. All these genes showed significant expression changes between 45 d and 100 d in HVC (Figure 3C). In the RNA‐Seq experiment, expression levels of these genes were represented with read counts ranging from 15 to several thousand, and fold‐changes from 1.7‐fold to 13‐fold. Despite these wide ranges and different analysis platforms, regression analysis indicated a high level correlation between the RNA‐Seq and RT‐qPCR results (*R* ^2^ = 0.8318, Figure 3B). We further performed RT‐qPCR to test eight differentially expressed genes that did not meet with the stringent *q* < 0.1 cutoff, but had *p* < 0.01 in the RNA‐Seq experiment. This second group, which included *CAMK2B‐1*, *CRIPT*, *FAM19A1*, *GABRB1‐2*, *GAD2*, *NEUROD6*, *RAPGEF1*, and *UBE2A*, also showed significant expression changes between 45 d and 100 d HVC by RT‐qPCR, and the RNA‐Seq and RT‐qPCR results showed a good correlation (*R* ^2^ = 0.69, Figure 3B,C. For all RT‐qPCR experiments, n = 4‐9 animals per age group were used. The RT‐qPCR data are also shown in Data S5). Together, these results provide an independent validation of our RNA‐Seq approach to identifying differentially expressed genes in HVC during song development.

**FIGURE 4:**
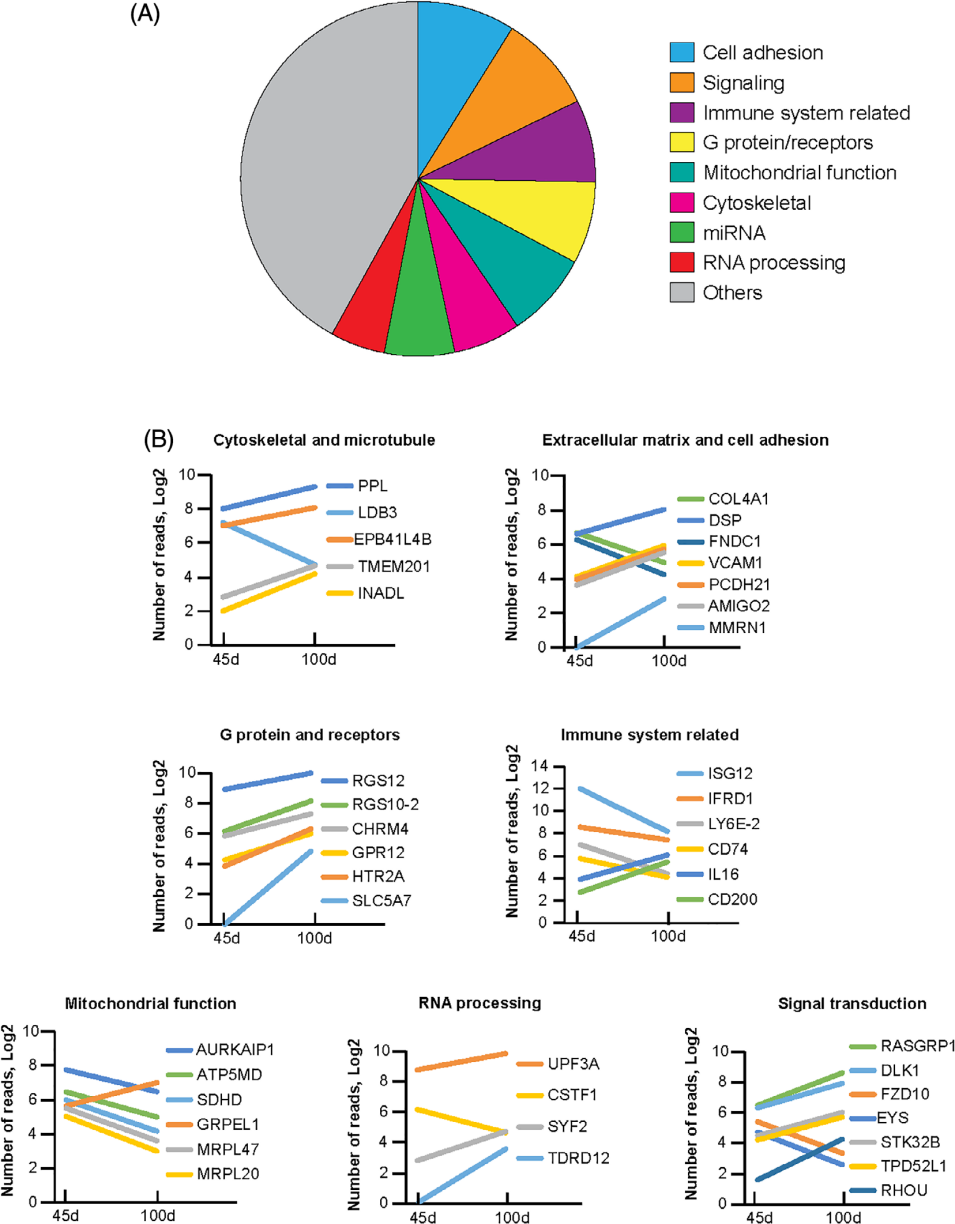
Functional groups of genes differentially expressed between juvenile and adult HVC. A, Relative distribution of functional groups of genes differentially expressed between 45 d and 100 d HVC. B, Functional groups of genes that changed expression significantly (*q* < 0.1): cytoskeleton and microtubules, extracellular matrix and cell adhesion, G protein and G‐protein‐coupled receptors, immune system related, mitochondrial function, RNA processing, and signal transduction. Each line connects the numbers of reads at 45 d and 100 d in HVC. Line colors identify gene names as indicated

To examine the functions of the developmentally regulated genes, they were sorted into groups according to their functional annotations. The relative distribution of these groups is summarized in Figure 4A. These groups include genes with functions related to cytoskeleton and microtubule, extracellular matrix and cell adhesion, G protein‐coupled neuromodulator receptors, immune system function, mitochondrial function, RNA processing, and signal transduction (Figure 4B). The cell adhesion and extracellular matrix proteins have important roles in neuronal migration and maturation, while the cytoskeletal and microtubule proteins represent essential structural components for establishing and modifying neuronal dendritic and synaptic morphology. Within these groups, some genes were upregulated and others downregulated between the two ages. Among the signal transduction genes, FZD10 is known to promote sensory neuron development in Xenopus^35^ and was observed here as upregulated at 45 d. In contrast, genes encoding proteins for synaptic functions, including G‐protein coupled neuromodulator receptors, were mostly upregulated at 100 d compared to 45 d. These patterns are consistent with and reflect the highly orchestrated processes of circuit maturation and synaptic reorganization, particularly, the transition from synaptogenesis at 45 d to mature synaptic functions as zebra finches mature into adulthood. The downregulation of genes related to mitochondrial function may indicate the shifting of energy demand during this process. Dysfunction of many of these genes has been associated with a wide range of neurodevelopmental disorders (Table 1).

**TABLE 1:** Genes developmentally regulated in HVC that have been implicated in nervous system related disorders

Gene symbol	Gene name	Disorders^a^
CLCN2	Chloride channel 2	Epilepsy
COL4A1	α1 chain of type IV collagen	Cerebral small vessel disease, epilepsy, developmental delay
CRIPT	CXXC repeat containing Interactor of PDZ3 domain	Microcephaly, developmental delay
IFRD1	Interferon related developmental regulator 1	Sensory/motor neuropathy with ataxia (SMNA)
RAPGEF1	Rap guanine nucleotide exchange factor 1	Cerebral palsy, communication difficulties, cognitive impairment
TLL2	Zinc‐dependent metalloprotease	Non‐Syndromic intellectual disability, ADHD
UBE2A	Ubiquitin‐conjugating enzyme E2A	Intellectual disability, seizure, poor speech
UPF3A	Regulator of nonsense mediated MRNA decay	ASDs

Abbreviation: ASDs, autism spectrum disorders.

^a^ Note all genes listed met with *q* < 0.1 cutoff in RNA‐Seq or have been validated by RT‐qPCR. Mutations of some of these genes have been implicated in multiple diseases, but only nervous system disorders are listed.

Unexpectedly, a group of genes known for their functions in the immune system was found as developmentally regulated in HVC (Table 2). This group includes genes encoding the major histocompatibility complex class II invariant chain1 (CD74), the immune inhibitory molecule CD200, the brain‐specific chemokine (FAM19A1), interferon‐related developmental regulator 11 (IFRD1), interleukin IL16, interferon inducible protein ISG12, and Lymphocyte antigen complex 6 (LY6E‐2). Expression changes of these genes were validated using RT‐qPCR (see Figure 3C,D). In addition, at least some of them (eg, *ISG12* and *LY6E‐2*) exhibited enriched expression specifically in HVC and other song related brain regions (Figure 1C). Together, these observations argue against the possibility that their expression patterns were due to pathogen‐activated immune responses, although that cannot be entirely excluded.

**TABLE 2:** Immune system related genes that are developmentally regulated in HVC

Gene symbol	Descriptive name	Log2 FC^a^
CD74	MHC class II invariant chain 1	−1.690
CD200	Type I membrane glycoprotein	2.720
FAM19A1	Brain‐specific chemokine	−0.863
IFRD1	Interferon‐related developmental regulator 1	−1.125
IL16	Interleukin 16	2.172
ISG12	Interferon inducible protein	−3.758
LY6E‐2	Lymphocyte antigen complex 6	−2.634

^a^ Note positive values indicate increased expression and negative values indicate decreased expression in HVC from 45 d to 100 d. The fold changes are based on RNA‐Seq results, all have been validated by RT‐qPCR with *p* < 0.05 (see Figure 3).

**FIGURE 5:**
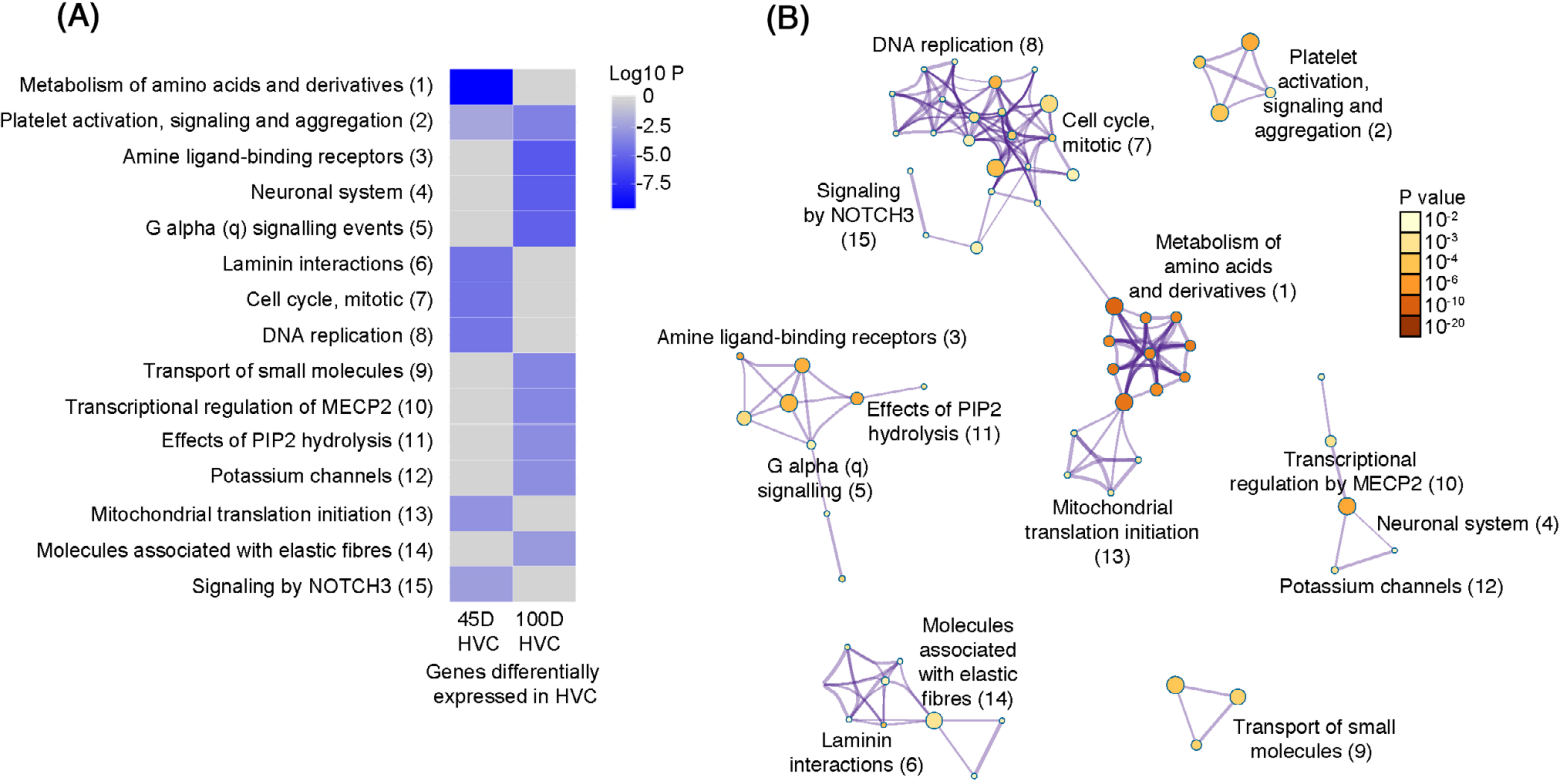
REACTOME enrichment analysis of genes differentially expressed in the HVC between 45 d and 100 d. A, Enriched REACTOME terms among genes showing higher expression in the HVC at 45 d or 100 d. B, Interconnections between differentially expressed terms in the HVC at 45 d or 100 d. In both A and B, *P* values are color‐coded; grey represents the absence of that term at the specified age

We performed REACTOME analysis of genes displaying developmentally regulated expression in HVC. The most highly enriched term among genes with higher expression in 45 d HVC is metabolism of amino acids and derivatives. This group includes many genes related to ribosomal proteins, proteasome proteins, and translation initiation and elongation. Interestingly, terms related to cell division such as DNA replication and repair, G1/S phase transition, cell cycle, and mitosis are highly enriched. Genes with functions related to laminin interactions such as extracellular matrix organization, integrin cell surface interactions, and crosslinking of collagen fibrils are enriched as well. Other enriched terms include mitochondrial protein synthesis. Network analysis further reveals interconnections among these terms (Figure 5A,B and Data S6). These interactions suggest that, instead of singular and independent events, well‐orchestrated and well‐coordinated gene expression changes collectively play critical roles during HVC development. As juveniles mature to adults, genes with higher expression in 100 d HVC are enriched with terms pertaining to neuronal system functions, these terms include genes encoding for calcium channels, potassium channels, sodium channels and glutamate transporters, various amine ligand binding receptors (including dopamine receptors, adrenergic receptors, and serotonin receptors), and G protein signaling. Other enriched terms include transport of small molecules, transcriptional regulation of MECP2, and effects of PIP2 hydrolysis. These changing enrichment patterns in HVC reflect orchestrated gene expression changes occurring as the growth and formation of circuit connections in juveniles switches to the mature functional state in adults.

## 3 DISCUSSION

These RNA sequencing based experiments have enabled us to generate a comprehensive genome‐wide inventory of gene expression in the song control nucleus HVC in juvenile and adult male zebra finches. While a large portion of the genome is active in HVC during its development, we noted that a sizable fraction of the high‐quality sequence reads (~40%) failed to map to the zebra finch genome. Of the reads mapped to the genome, a large fraction mapped to regions without annotation. The incomplete assembly and annotation of the zebra finch genome may have contributed to these observations. However, in recent years, as the sensitivity of detection techniques improves, mounting evidence has indicated that, in addition to known genes, a large portion of mammalian genomes is transcriptionally active. This phenomenon, now known as “pervasive transcription,” could give rise to long non‐coding, intronic, intergenic, and/or antisense transcripts.^36^, ^37^ The biological functions of these novel “dark matter” transcripts have yet to be explored. Our data add evidence to the suggestion that similar pervasive transcription occurs from non‐annotated regions of the zebra finch genome as well.

The RNA‐Seq results reveal complex and dynamic gene expression profiles in HVC of juvenile and adult zebra finches. Although gene expression patterns in HVC at 45 d and 100 d share many similarities, they are both clearly distinct from the remainders of the brain. Many genes with general cellular functions are highly enriched in juvenile HVC. For example, gene pathways related to electron transport and ATP synthesis are highly expressed in 45 d HVC, highlighting the energy metabolism demand at this age. A large number of genes related to transcription and RNA processing are also highly enriched in 45 d HVC. These groups include components for RNA polymerase, mRNA splicing, cleavage of growing transcript in the termination region, and mRNA 3′‐end processing. Many neuronal genes use alternative splicing to increase gene product diversity, and via regulating gene products and functions, RNA splicing can affect many aspects of neural development and physiology.^38^, ^39^ In mammalian visual cortex, Benoit et al report that many genes are differentially regulated, but many more splicing isoforms are differentially regulated during the critical period, suggesting the importance of posttranscriptional regulation and RNA splicing in critical period plasticity.^40^ Our observation of highly enriched expression of RNA processing‐related genes reflects the high transcriptional activity in juvenile HVC, and suggests that posttranscriptional regulatory events likely play a role in defining the structural and functional state of HVC.

RNA‐Seq data show that, as juveniles mature into adulthood, massive changes in gene expression landscape occur in HVC, indicating that at the postnatal age of 45 d, development of the song circuit is far from complete. HVC does not reach its adult size until well past 45 d.^19^ It is known that cell proliferation occurs in the ventricular zone (VZ) lying on the top of HVC. The new cells migrate a short distance into HVC, and differentiate into neurons.^41^, ^42^ A large number of new neurons are born during post‐hatching day 30 to day 65, and are incorporated into the HVC circuit.^20^, ^43^ We observe that genes related to DNA replication and cell proliferation are among those with higher expression in 45 d HVC. It is possible that the HVC samples might have included some VZ tissues since it is difficult to separate VZ from HVC during tissue dissection. It is also possible that cell types other than neurons, such as oligodendrocytes or astrocytes, are proliferating in HVC. Recently, Anda et al report that during mammalian brain development (eg, P3), new neurons in the cortical plate that have just migrated from VZ express many cell cycle related genes, and have the potential of proliferation.^44^ Further investigation could answer whether a similar process occurs in the avian cortical nucleus HVC.

The highly enriched expression of genes related to axon guidance and growth in 45 d HVC supports the morphological and functional changes associated with innervation of HVC projection fibers after juvenile zebra finches begin to sing. Although singing begins at about 30 d, HVC projection fibers only begin to innervate and make synaptic connections with the downstream nucleus RA around postnatal day 35,^18^ and at about 45 d, song control transitions from lMAN to HVC.^16^ Many genes pertaining to extracellular adhesion and Slit‐ROBO signaling, typically acting via ligand‐receptor interactions, have important roles in neuron migration, axon guidance, cell‐cell and cell‐matrix recognition, and establishing synaptic connections.^45^ Members of the Split‐ROBO signaling pathway have been shown to be expressed in HVC and to be developmentally regulated in RA, the downstream nucleus to which HVC connects.^25^, ^46^ Other members of the axon guidance term enriched in 45 d HVC include genes coding for calcium channel, potassium channel, NMDA receptors, and endocytosis proteins. We have noted that many genes enriched in 45 d HVC are also enriched in adult HVC, but to a lesser extent. Their continued expression in adult HVC, reported as well by Lovell et al,^25^ is expected, since many of these signaling molecules (eg, Slit‐ROBO), channels, and receptors are the very components that define neuronal properties and functions.

As HVC matures, enriched gene expression shifts to components of mature functional neural circuits. We noted that a group of genes related to retinoid metabolism are enriched in adult HVC, a complement to previous findings that *ALDH1A2* plays critical roles in HVC function and song learning.^33^, ^47^, ^48^ By analyzing earlier cDNA microarray data, Lovell et al report a large number of genes differentially expressed in adult HVC compared to the adjacent shelf.^24^, ^25^ Although that study and the present study used different methods (cDNA microarray vs RNA‐Seq) and different references for gene comparison (HVC vs shelf opposed to HVC vs whole brain tissue), the two studies share many concordant observations. For example, both find enriched expression of potassium channel genes in HVC. These findings, together with an earlier study reporting a large number of potassium channel genes in HVC,^49^ strengthen the roles potassium channels play in defining the complex electrical firing properties of HVC neurons, whose bursting patterns are linked to the temporal pattern of a song.^17^ Another common theme between the two studies is the enriched expression of genes related to G protein signaling and many receptors for neural modulators in adult HVC. The latter group includes adrenergic receptors, dopamine receptors, serotonin receptors, and cholinergic receptors. These diverse modulatory mechanisms of G protein‐coupled receptors provide many possibilities for HVC structure and function to be tuned in response to physiological and environmental events. The massive gene expression change during extended postnatal development is not limited to HVC. London et al have shown that gene expression in auditory regions of zebra finches at 20 d is vastly different from that in adults, even though the gross morphology of these regions does not significantly differ between juveniles and adults.^26^ It appears that age plays important roles in regulating gene expression in song related brain regions, and the extended period of development would allow the structure and function of these brain regions to be modulated by sensory‐motor and/or perceptual learning experience via gene expression changes.

Some genes developmentally regulated in HVC have been implicated in neural developmental disorders. *COL4A1* encodes collagen type IV α1 chain, an extracellular matrix protein. Mutations in *COL4A1* have been found to cause cerebral vasculature defects, migraine, stroke, and epilepsy.^50^, ^51^ Other members of the collagen family, *COL12A1* and *COL21A1*, are also developmentally regulated in HVC,^29^ highlighting the roles of this gene family in HVC development. *RAFGEF1* encodes Rap guanine nucleotide exchange factor 1, which modulates GTPase signaling pathways. *RAFGEF1* has been linked to Autism Spectrum Disorders (ASDs), cerebral palsy, communication difficulties, and cognitive impairments.^52^, ^53^ *TLL2* encodes the zinc‐dependent metalloprotease tolloid‐like protein 2, a protease required for organizing extracellular matrix. Diseases associated with *TLL2* include autosomal recessive non‐syndromic intellectual disability.^54^, ^55^ *UBE2A* encodes a member of the E2 ubiquitin‐conjugating enzyme family, which catalyzes the covalent attachment of ubiquitin to substrate proteins. Disorders associated with *UBE2A* include mental retardation, seizures, poor speech, and aggressive behavior.^56^, ^57^, ^58^ *UPF3A* encodes the regulator of nonsense mediated mRNA decay (NMD), a process cells use to eliminate faulty mRNAs. UPF3A and its paralog UPF3B antagonistically regulate NMD, and both have been implicated in ASDs, intellectual disability, and schizophrenia.^59^, ^60^, ^61^, ^62^ The links between these genes and neurodevelopmental disorders are often established by genome‐wide screening for mutations and/or copy number variations in human patients. Further investigation of these genes in a neural circuit for vocal communication will likely provide insight into the molecular and cellular mechanisms underlying these developmental disorders in humans.

It is puzzling that a group of genes known to function in immune regulation and inflammatory responses are developmentally regulated in HVC. The role they may play in HVC is unclear. Emerging research has implicated microglia in regulating neurogenesis and neuronal cell numbers during neural development by initiating neuronal apoptosis followed by cleaning cellular debris through phagocytosis.^63^ The phagocytic microglia also modulate wiring of the neural circuits by regulating the number, maturation, and plasticity of synaptic connections.^64^, ^65^ Recent studies have revealed that many microglia functions are mediated by components of the classical complement cascade or cytokine signaling pathways. For example, the complement cascade components C1q and C3 as well as C3 receptor (CR3) have been shown to mediate synaptic pruning in the developing retinogeniculate system in an activity‐dependent manner.^66^, ^67^, ^68^ Similarly, neuronal expression of MHC Class I genes has been implicated in activity‐dependent regulation of ocular dominance plasticity during the critical period of visual system development.^69^, ^70^ During song circuit development, extensive neurogenesis and neuronal cell death as well as formation and modification of synaptic connections occur in HVC.^15^, ^20^ The regulation of major histocompatibility complex class II invariant chain (CD74) and various cytokines and their receptors (eg, FAM19A1 and IL16) in HVC suggests that they may play a role in the development and maturation of HVC. The HVC tissue we sequenced presumably contained multiple cell types, including projection neurons, interneurons, glia, and microglia. Each of these cell types could have contributed to the gene expression profiles. Further cell type specific gene expression analysis will help delineate in which cell types these genes express, and help understand their contribution to HVC development.

The RNA‐Seq data obtained in this study provide new insights into the molecular and genomic underpinning in HVC as juveniles mature to adulthood. The datasets will also join other resources to benefit future studies investigating functions of individual genes and/or interactions among genes during song circuit development and vocal learning in songbird. RNA‐seq, with its high throughput capacity, higher sensitivity, and unbiased nature, has increasingly become a standard approach to large scale quantitative gene expression analysis, and allows for cross comparison of data from different studies, from different laboratories, and even from different animal species. As exploration of transcriptomes has become an important approach to understanding circuit development and brain diseases, these datasets can serve as a reference for further transcriptome analysis relevant to a wide range of physiological and/or experimental conditions.

## 4 METHODS

### 4.1 Animals, tissue collection, and RNA isolation

Animal usage was approved by the Louisiana State University Health School of Medicine Institutional Animal Care and Use Committee. The juvenile and adult zebra finches were obtained from 10‐15 breeding cages in our breeding colony at the LSU School of Medicine animal care facility with birth dates documented. The juveniles were raised with their parents in family cages until 45 days of age. To ensure that gene expression patterns reflect developmental stages, and were not confounded by behavioral factors (eg, singing), birds were monitored in the morning for one hour individually and brains were collected from birds that did not sing and did not hear songs. For HVC tissue collection, birds were euthanized at a specified age, their brains were collected and snap frozen in liquid nitrogen or dry ice. Brains were cut into 80 μm sagittal sections, HVC tissue was dissected out using a syringe needle under a dissection microscope, and quickly transferred into a lysis buffer for RNA isolation. We tried not to include the adjacent tissue on the medial/lateral and ventral sides of HVC. The ventricular zone lies immediately on the top of HVC, and it is difficult to separate it from HVC. Thus, the HVC samples may have contained portions of the ventricular zone. We stained sections especially of juveniles to define the medial/lateral edges of HVC. If there was even a slight doubt, that section was not used. Total RNA was isolated using Trizol reagents (Invitrogen). Residual contaminating genomic DNA was removed by DNAse I digestion. RNA concentration was determined using the Nanodrop spectrometer, and RNA quality was examined using the Agilent Bioanalyzer.

### 4.2 cDNA library preparation and sequencing

Total RNAs from HVC of four birds per age group were pooled and prepared into one sequencing library using a NuGEN Ovation RNAseq V1 kit following the manufacturer's instructions. An additional step of S1 nuclease treatment of cDNA was used as described.^71^ Sequencing libraries were size selected on a 2% agarose gel to obtain libraries in a size range of 350 to 400 bp corresponding to inserts lengths of 230 to 280 bp. Libraries were also made from whole brain tissues of male zebra finches at 45 d and 100 d by pooling two brains per library. We sequenced each library on an Illumina Genome Analyzer II (GAIIx) sequencer, which produced ~20 million sequence reads of 79 nt per lane. Library construction and high‐throughput sequencing were performed by the Next Generation Sequencing core, The Scripps Research Institute. For more detailed methods description, see.^71^

### 4.3 RNA‐Seq analysis

We used Casava 1.7 for demultiplexing and base calling to generate FASTQ files containing the sequence reads. After quality filtering and adaptor trimming, high quality reads were mapped to the zebra finch genome (3.2.4/taeGut1) using the ELAND2 (Illumina) software to define gene structure and alternative splicing events, and to quantify transcript abundance. We used R Bioconductor package EdgeR to compare gene expression between the two age groups. EdgeR provides statistical routines for digital gene expression data and ranks genes by statistical significance, taking into account the total read number, sample reproducibility, and fold changes. Comparing the normalized read frequencies allowed identification of genes with enriched expression in HVC and genes regulated in HVC during development.

### 4.4 REACTOME pathway enrichment analysis

Gene lists were constructed as follows: For comparing HVC and WB at 45 d and 100 d, HVC enriched (*p* < 0.01) gene symbols were extracted from EdgeR output and merged into one file with two separate columns: 45 d HVC‐enriched and 100 d HVC‐enriched. Similarly, for comparing 45 d and 100 d HVC samples, upregulated (*p* < 0.05) gene symbols were extracted from EdgeR output and merged into one file with two columns: 45 d HVC and 100 d HVC. The gene lists were subjected to Metascape^72^ for REACTOME enrichment analysis in CUSTOM option with default parameters. Note, since the REACTOME database does not include zebra finch genes, human orthologous genes were used as background in this analysis. Pathway terms with *p* < 0.01 were considered significant. Network analysis was also performed using Metascape in CUSTOM option with default parameters, and the resulting layouts were manually modified with Cytoscape (3.3.0) using approaches as described in References 73, 74.

### 4.5 RT‐qPCR

RT‐qPCR was performed as described previously.^75^ Briefly, total RNA was isolated from HVC tissue using Trizol reagent (Invitrogen), and quantified using a Nanodrop spectrophotometer. Reverse transcription was performed using 50 ng of total RNA using an iScript Reverse Transcription Supermix kit (Bio‐Rad) following the manufacturer's instructions. qPCR was performed using the iQ SYBR Green Supermix (Bio‐Rad) following the manufacturer's instructions. *GAPDH* was used as a reference gene after determining that its expression did not change during development. Relative gene expression levels were determined using the comparative Ct (2^−ΔΔCt^) method after normalizing to *GAPDH*.^76^ N = 4‐9 animals per age group were used in the RT‐qPCR experiment. For all samples, RT‐qPCR was performed in triplicate twice. Results shown in Figure 3 and Data S5 are from one set of experiment. Dissociation curve analysis was performed to confirm a single peak PCR product for each gene, indicating the specificity of PCR reactions. All primers were obtained from IDT (Integrated DNA Technology); their sequences are listed in Data S7.

### 4.6 In situ hybridization

In situ hybridization was performed as described previously.^34^ Briefly, fresh frozen zebra finch brains were cut into 10 μm sagittal sections and kept at −80°C until use. Brain sections were fixed in 4% PFA for 10 minutes followed by acetylation for 10 minutes. PCR amplified cDNA fragments containing the probe sequences (200‐300 bp, sequence‐verified) were cloned into pBluescript plasmid vectors, and sequences were verified. ^33^P‐labeled ribo‐probes were made by in vitro transcription using T7 or T3 RNA polymerase (PerkinElmer Kit). For hybridization, 10^6^ cpm probes in 40 μL hybridization buffer were added to each brain section and hybridized at 65°C overnight. After hybridization, brain sections were washed two times, 30 minutes each, at 65°C with wash solution (50% formamide, 1xSSC, and 0.1% Tween 20), followed by washing 2 times with 0.2 X SSC at 65°C. Slides were exposed to X‐films for 1‐7 days, depending on signal intensity.

## AUTHOR CONTRIBUTIONS

**Zhimin Shi**: Data collection, analysis, and manuscript preparation; **Zeyu Zhang**: Data analysis and manuscript preparation; **Lana Schaffer**: Data analysis; **Zhi Huang**: Data collection; **Lijuan Fu**: Data collection; **Steven Head**: data collection; **Terry Gaasterland**: Experimental design, data analysis, and manuscript preparation; **Xiu‐Jie Wang**: Experimental design, data analysis and manuscript preparation; **XiaoChing Li**: Experimental design, data analysis, manuscript preparation, and project management.

## CONFLICT OF INTEREST

All authors declare no conflict of interest.

## DATA AVAILABILITY STATEMENT

The RNA‐Seq data are available in the *Gene Expression Omnibus* (*GEO*) repository under accession number GSE140470 https://www.ncbi.nlm.nih.gov/geo/query/acc.cgi?acc=GSE140470.

## Supporting information


**Table S1** Summary of sequencing and mapping results

**Data S1** Comparing gene expression between HVC and whole brain samples at 45 d of age

**Data S2** Comparing gene expression between HVC and whole brain samples at 100 d

**Data S3** REACTOME enrichment analysis of HVC‐enriched genes at 45 d and 100 d

**Data S4** Comparing gene expression between 45 d and 100 d HVC

**Data S5** RT‐qPCR validation of RNA‐Seq results

**Data S6** REACTOME enrichment analysis of upregulated genes in 45 d or 100 d HVC samples

**Data S7** Sequences of RT‐qPCR primes

## References

[1] Zann R . The Zebra Finch. New York, NY: Oxford University Press; 1996.

[2] Immelmann K . Song development in zebra finch and other Estrildid finches. In: Hinde R , ed. Bird Vocalisations. London, UK: Cambridge University Press; 1969.

[3] Doupe AJ , Kuhl PK . Birdsong and human speech: common themes and mechanisms. Annu Rev Neurosci. 1999;22:567‐631. PubMed PMID: 10202549. 10202549 10.1146/annurev.neuro.22.1.567

[4] Tchernichovski O , Mitra PP , Lints T , Nottebohm F . Dynamics of the vocal imitation process: how a zebra finch learns its song. Science. 2001;291(5513):2564‐2569. PubMed PMID: 11283361. 11283361 10.1126/science.1058522

[5] Roberts TF , Mooney R . Motor circuits help encode auditory memories of vocal models used to guide vocal learning. Hear Res. 2013;303:48‐57. PubMed PMID: 23353871. Pubmed Central PMCID: 3689868. 23353871 10.1016/j.heares.2013.01.009 PMC3689868

[6] Roper A , Zann R . The onset of song learning and song tutor selection in fledgling zebra finches. Ethology. 2006;112(5):458‐470. PubMed PMID: WOS:000236799000005. English.

[7] Bottjer SW , Miesner EA , Arnold AP . Forebrain lesions disrupt development but not maintenance of song in passerine birds. Science. 1984;224(4651):901‐903. PubMed PMID: 6719123. 6719123 10.1126/science.6719123

[8] Bottjer SW , Johnson F . Circuits, hormones, and learning: vocal behavior in songbirds. J Neurobiol. 1997;33(5):602‐618. PubMed PMID: 9369462. 9369462 10.1002/(sici)1097-4695(19971105)33:5<602::aid-neu8>3.0.co;2-8

[9] Scharff C , Nottebohm F . A comparative study of the behavioral deficits following lesions of various parts of the zebra finch song system: implications for vocal learning. J Neurosci. 1991;11(9):2896‐2913. PubMed PMID: 1880555. 1880555 10.1523/JNEUROSCI.11-09-02896.1991 PMC6575264

[10] Simpson HB , Vicario DS . Brain pathways for learned and unlearned vocalizations differ in zebra finches. J Neurosci. 1990;10(5):1541‐1556. PubMed PMID: 2332796. 2332796 10.1523/JNEUROSCI.10-05-01541.1990 PMC6570078

[11] Nottebohm F , Stokes TM , Leonard CM . Central control of song in the canary, Serinus canarius. J Comp Neurol. 1976;165(4):457‐486. PubMed PMID: 1262540. 1262540 10.1002/cne.901650405

[12] Vates GE , Broome BM , Mello CV , Nottebohm F . Auditory pathways of caudal telencephalon and their relation to the song system of adult male zebra finches. J Comp Neurol. 1996;366(4):613‐642. PubMed PMID: 8833113. 8833113 10.1002/(SICI)1096-9861(19960318)366:4<613::AID-CNE5>3.0.CO;2-7

[13] Nottebohm F . The anatomy and timing of vocal learning in birds. In: Hauser MD , Konishi M , eds. The Design of Animal Communication. Cambridge, MA: MIT Press; 1999:63‐110.

[14] Hahnloser RH , Kozhevnikov AA , Fee MS . An ultra‐sparse code underlies the generation of neural sequences in a songbird. Nature. 2002;419(6902):65‐70. PubMed PMID: 12214232. 12214232 10.1038/nature00974

[15] Roberts TF , Tschida KA , Klein ME , Mooney R . Rapid spine stabilization and synaptic enhancement at the onset of behavioural learning. Nature. 2010;463(7283):948‐952. PubMed PMID: 20164928. Pubmed Central PMCID: 2918377. 20164928 10.1038/nature08759 PMC2918377

[16] Aronov D , Andalman AS , Fee MS . A specialized forebrain circuit for vocal babbling in the juvenile songbird. Science. 2008;320(5876):630‐634. PubMed PMID: 18451295. 18451295 10.1126/science.1155140

[17] Long MA , Fee MS . Using temperature to analyse temporal dynamics in the songbird motor pathway. Nature. 2008;456(7219):189‐194. PubMed PMID: 19005546. Pubmed Central PMCID: 2723166. 19005546 10.1038/nature07448 PMC2723166

[18] Mooney R , Rao M . Waiting periods versus early innervation: the development of axonal connections in the zebra finch song system. J Neurosci. 1994;14(11 Pt 1):6532‐6543. PubMed PMID: 7965057. Pubmed Central PMCID: 6577238. 7965057 10.1523/JNEUROSCI.14-11-06532.1994 PMC6577238

[19] Alvarez‐Buylla A , Ling CY , Nottebohm F . High vocal center growth and its relation to neurogenesis, neuronal replacement and song acquisition in juvenile canaries. J Neurobiol. 1992;23(4):396‐406. PubMed PMID: 1634887. 1634887 10.1002/neu.480230406

[20] Wilbrecht L , Williams H , Gangadhar N , Nottebohm F . High levels of new neuron addition persist when the sensitive period for song learning is experimentally prolonged. J Neurosci. 2006;26(36):9135‐9141. PubMed PMID: 16957070. 16957070 10.1523/JNEUROSCI.4869-05.2006 PMC6674498

[21] Tomaszycki ML , Peabody C , Replogle K , Clayton DF , Tempelman RJ , Wade J . Sexual differentiation of the zebra finch song system: potential roles for sex chromosome genes. BMC Neurosci. 2009;10:24 PubMed PMID: 19309515. Pubmed Central PMCID: 2664819. 19309515 10.1186/1471-2202-10-24 PMC2664819

[22] Drnevich J , Replogle KL , Lovell P , et al. Impact of experience‐dependent and ‐independent factors on gene expression in songbird brain. Proc Natl Acad Sci U S A. 2012;109(SUPPL 2):17245‐17252. PubMed PMID: 23045667. Pubmed Central PMCID: 3477375. 23045667 10.1073/pnas.1200655109 PMC3477375

[23] Pfenning AR , Hara E , Whitney O , et al. Convergent transcriptional specializations in the brains of humans and song‐learning birds. Science. 2014;346(6215):1256846 PubMed PMID: 25504733. Pubmed Central PMCID: 4385736. 25504733 10.1126/science.1256846 PMC4385736

[24] Lovell PV , Clayton DF , Replogle KL , Mello CV . Birdsong "transcriptomics": neurochemical specializations of the oscine song system. PLoS One. 2008;3(10):e3440 PubMed PMID: 18941504. Pubmed Central PMCID: 2563692. 18941504 10.1371/journal.pone.0003440 PMC2563692

[25] Lovell PV , Huizinga NA , Friedrich SR , Wirthlin M , Mello CV . The constitutive differential transcriptome of a brain circuit for vocal learning. BMC Genomics. 2018;19(1):231 PubMed PMID: 29614959. Pubmed Central PMCID: 5883274. 29614959 10.1186/s12864-018-4578-0 PMC5883274

[26] London SE , Dong S , Replogle K , Clayton DF . Developmental shifts in gene expression in the auditory forebrain during the sensitive period for song learning. Dev Neurobiol. 2009;69(7):437‐450. PubMed PMID: 19360720. Pubmed Central PMCID: 2765821. 19360720 10.1002/dneu.20719 PMC2765821

[27] Wada K , Howard JT , McConnell P , et al. A molecular neuroethological approach for identifying and characterizing a cascade of behaviorally regulated genes. Proc Natl Acad Sci U S A. 2006;103(41):15212‐15217. PubMed PMID: 17018643. Pubmed Central PMCID: 1622802. 17018643 10.1073/pnas.0607098103 PMC1622802

[28] Whitney O , Pfenning AR , Howard JT , et al. Core and region‐enriched networks of behaviorally regulated genes and the singing genome. Science. 2014;346(6215):1256780 PubMed PMID: 25504732. Pubmed Central PMCID: 4359888. 25504732 10.1126/science.1256780 PMC4359888

[29] Olson CR , Hodges LK , Mello CV . Dynamic gene expression in the song system of zebra finches during the song learning period. Dev Neurobiol. 2015;75(12):1315‐1338. PubMed PMID: 25787707. Pubmed Central PMCID: 4575259. 25787707 10.1002/dneu.22286 PMC4575259

[30] Kelly TK , Ahmadiantehrani S , Blattler A , London SE . Epigenetic regulation of transcriptional plasticity associated with developmental song learning. Proc Biol Sci. 2018;285:20180160. PubMed PMID: 29720411. Pubmed Central PMCID: 5966592. 29720411 10.1098/rspb.2018.0160 PMC5966592

[31] Mello CV , Vicario DS , Clayton DF . Song presentation induces gene expression in the songbird forebrain. Proc Natl Acad Sci U S A. 1992;89(15):6818‐6822. PubMed PMID: 1495970. Pubmed Central PMCID: 49595. 1495970 10.1073/pnas.89.15.6818 PMC49595

[32] Jarvis ED , Nottebohm F . Motor‐driven gene expression. Proc Natl Acad Sci U S A. 1997;94(8):4097‐4102. PubMed PMID: 9108111. Pubmed Central PMCID: 20574. 9108111 10.1073/pnas.94.8.4097 PMC20574

[33] Denisenko‐Nehrbass NI , Jarvis E , Scharff C , Nottebohm F , Mello CV . Site‐specific retinoic acid production in the brain of adult songbirds. Neuron. 2000;27(2):359‐370. PubMed PMID: 10985355. 10985355 10.1016/s0896-6273(00)00043-x

[34] Li X , Wang XJ , Tannenhauser J , et al. Genomic resources for songbird research and their use in characterizing gene expression during brain development. Proc Natl Acad Sci U S A. 2007;104(16):6834‐6839. PubMed PMID: 17426146. Pubmed Central PMCID: 1850020. 17426146 10.1073/pnas.0701619104 PMC1850020

[35] Garcia‐Morales C , Liu CH , Abu‐Elmagd M , Hajihosseini MK , Wheeler GN . Frizzled‐10 promotes sensory neuron development in Xenopus embryos. Dev Biol. 2009;335(1):143‐155. PubMed PMID: 19716814. 19716814 10.1016/j.ydbio.2009.08.021

[36] Armour CD , Castle JC , Chen R , et al. Digital transcriptome profiling using selective hexamer priming for cDNA synthesis. Nat Methods. 2009;6(9):647‐649. PubMed PMID: 19668204. 19668204 10.1038/nmeth.1360

[37] Clark MB , Amaral PP , Schlesinger FJ , et al. The reality of pervasive transcription. PLoS Biol. 2011;9(7):e1000625 discussion e1102. PubMed PMID: 21765801. Pubmed Central PMCID: 3134446. 21765801 10.1371/journal.pbio.1000625 PMC3134446

[38] Raj B , Blencowe BJ . Alternative splicing in the mammalian nervous system: recent insights into mechanisms and functional roles. Neuron. 2015;87(1):14‐27. PubMed PMID: 26139367. 26139367 10.1016/j.neuron.2015.05.004

[39] Vuong CK , Black DL , Zheng S . The neurogenetics of alternative splicing. Nat Rev Neurosci. 2016;17(5):265‐281. PubMed PMID: 27094079. Pubmed Central PMCID: 4861142. 27094079 10.1038/nrn.2016.27 PMC4861142

[40] Benoit J , Ayoub AE , Rakic P . Transcriptomics of critical period of visual cortical plasticity in mice. Proc Natl Acad Sci U S A. 2015;112(26):8094‐8099. PubMed PMID: 26080443. Pubmed Central PMCID: 4491803. 26080443 10.1073/pnas.1509323112 PMC4491803

[41] Alvarez‐Buylla A , Kirn JR , Nottebohm F . Birth of projection neurons in adult avian brain may be related to perceptual or motor learning. Science. 1990;249(4975):1444‐1446. PubMed PMID: 1698312. 1698312 10.1126/science.1698312

[42] Brenowitz EA , Larson TA . Neurogenesis in the adult avian song‐control system. Cold Spring Harb Perspect Biol. 2015;7:a019000. PubMed PMID: 26032719. Pubmed Central PMCID: 4448602. 26032719 10.1101/cshperspect.a019000 PMC4448602

[43] Nordeen KW , Nordeen EJ . Projection neurons within a vocal motor pathway are born during song learning in zebra finches. Nature. 1988;334(6178):149‐151. PubMed PMID: 3386754. 3386754 10.1038/334149a0

[44] Anda FC , Madabhushi R , Rei D , et al. Cortical neurons gradually attain a post‐mitotic state. Cell Res. 2016;26(9):1033‐1047. PubMed PMID: 27325298. Pubmed Central PMCID: 5034108. 27325298 10.1038/cr.2016.76 PMC5034108

[45] Blockus H , Chedotal A . Slit‐Robo signaling. Development. 2016;143(17):3037‐3044. PubMed PMID: 27578174. 27578174 10.1242/dev.132829

[46] Wang R , Chen CC , Hara E , et al. Convergent differential regulation of SLIT‐ROBO axon guidance genes in the brains of vocal learners. J Comp Neurol. 2015;523(6):892‐906. PubMed PMID: 25424606. Pubmed Central PMCID: 4329046. 25424606 10.1002/cne.23719 PMC4329046

[47] Olson CR , Rodrigues PV , Jeong JK , Prahl DJ , Mello CV . Organization and development of zebra finch HVC and paraHVC based on expression of zRalDH, an enzyme associated with retinoic acid production. J Comp Neurol. 2011;519(1):148‐161. PubMed PMID: 21120932. Pubmed Central PMCID: 3064427. 21120932 10.1002/cne.22510 PMC3064427

[48] Roeske TC , Scharff C , Olson CR , Nshdejan A , Mello CV . Long‐distance retinoid signaling in the zebra finch brain. PLoS One. 2014;9(11):e111722 PubMed PMID: 25393898. Pubmed Central PMCID: 4230966. 25393898 10.1371/journal.pone.0111722 PMC4230966

[49] Lovell PV , Carleton JB , Mello CV . Genomics analysis of potassium channel genes in songbirds reveals molecular specializations of brain circuits for the maintenance and production of learned vocalizations. BMC Genomics. 2013;14:470 PubMed PMID: 23845108. Pubmed Central PMCID: 3711925. 23845108 10.1186/1471-2164-14-470 PMC3711925

[50] Markus HS , Schmidt R . Genetics of vascular cognitive impairment. Stroke. 2019;50(3):765‐772. PubMed PMID: 30661498. Pubmed Central PMCID: 6420146. 30661498 10.1161/STROKEAHA.118.020379 PMC6420146

[51] Zagaglia S , Selch C , Nisevic JR , et al. Neurologic phenotypes associated with COL4A1/2 mutations: expanding the spectrum of disease. Neurology. 2018;91(22):e2078‐e2088. PubMed PMID: 30413629. Pubmed Central PMCID: 6282239. 30413629 10.1212/WNL.0000000000006567 PMC6282239

[52] de Ligt J , Willemsen MH , van Bon BW , et al. Diagnostic exome sequencing in persons with severe intellectual disability. N Engl J Med. 2012;367(20):1921‐1929. PubMed PMID: 23033978. 23033978 10.1056/NEJMoa1206524

[53] Oskoui M , Gazzellone MJ , Thiruvahindrapuram B , et al. Clinically relevant copy number variations detected in cerebral palsy. Nat Commun. 2015;6:7949 PubMed PMID: 26236009. Pubmed Central PMCID: 4532872. 26236009 10.1038/ncomms8949 PMC4532872

[54] Lesch KP , Timmesfeld N , Renner TJ , et al. Molecular genetics of adult ADHD: converging evidence from genome‐wide association and extended pedigree linkage studies. J Neural Transm. 2008;115(11):1573‐1585. PubMed PMID: 18839057. 18839057 10.1007/s00702-008-0119-3

[55] de Mooij‐van Malsen JG , van Lith HA , Laarakker MC , et al. Cross‐species genetics converge to TLL2 for mouse avoidance behavior and human bipolar disorder. Genes Brain Behav. 2013;12(6):653‐657. PubMed PMID: 23777486. 23777486 10.1111/gbb.12055

[56] Budny B , Badura‐Stronka M , Materna‐Kiryluk A , et al. Novel missense mutations in the ubiquitination‐related gene UBE2A cause a recognizable X‐linked mental retardation syndrome. Clin Genet. 2010;77(6):541‐551. PubMed PMID: 20412111. 20412111 10.1111/j.1399-0004.2010.01429.x

[57] Haddad DM , Vilain S , Vos M , et al. Mutations in the intellectual disability gene Ube2a cause neuronal dysfunction and impair parkin‐dependent mitophagy. Mol Cell. 2013;50(6):831‐843. PubMed PMID: 23685073. 23685073 10.1016/j.molcel.2013.04.012

[58] Tsurusaki Y , Ohashi I , Enomoto Y , et al. A novel UBE2A mutation causes X‐linked intellectual disability type Nascimento. Hum Genome Variation. 2017;4:17019 PubMed PMID: 28611923. Pubmed Central PMCID: 5462939. 10.1038/hgv.2017.19 PMC5462939 28611923

[59] Johnson MR , Shkura K , Langley SR , et al. Systems genetics identifies a convergent gene network for cognition and neurodevelopmental disease. Nat Neurosci. 2016;19(2):223‐232. PubMed PMID: 26691832. 26691832 10.1038/nn.4205

[60] Tarpey PS , Raymond FL , Nguyen LS , et al. Mutations in UPF3B, a member of the nonsense‐mediated mRNA decay complex, cause syndromic and nonsyndromic mental retardation. Nat Genet. 2007;39(9):1127‐1133. PubMed PMID: 17704778. Pubmed Central PMCID: 2872770. 17704778 10.1038/ng2100 PMC2872770

[61] Shum EY , Jones SH , Shao A , et al. The antagonistic gene paralogs Upf3a and Upf3b govern nonsense‐mediated RNA decay. Cell. 2016;165(2):382‐395. PubMed PMID: 27040500. Pubmed Central PMCID: 4826573. 27040500 10.1016/j.cell.2016.02.046 PMC4826573

[62] Linder B , Fischer U , Gehring NH . mRNA metabolism and neuronal disease. FEBS Lett. 2015;589(14):1598‐1606. PubMed PMID: 25957814. 25957814 10.1016/j.febslet.2015.04.052

[63] Thion MS , Ginhoux F , Garel S . Microglia and early brain development: an intimate journey. Science. 2018;362(6411):185‐189. PubMed PMID: 30309946. 30309946 10.1126/science.aat0474

[64] Schafer DP , Stevens B . Synapse elimination during development and disease: immune molecules take centre stage. Biochem Soc Trans. 2010;38(2):476‐481. PubMed PMID: 20298206. 20298206 10.1042/BST0380476

[65] Schafer DP , Heller CT , Gunner G , et al. Microglia contribute to circuit defects in Mecp2 null mice independent of microglia‐specific loss of Mecp2 expression. eLife. 2016;5:e15224. PubMed PMID: 27458802. Pubmed Central PMCID: 4961457. 27458802 10.7554/eLife.15224 PMC4961457

[66] Stevens B , Allen NJ , Vazquez LE , et al. The classical complement cascade mediates CNS synapse elimination. Cell. 2007;131(6):1164‐1178. PubMed PMID: 18083105. 18083105 10.1016/j.cell.2007.10.036

[67] Schafer DP , Lehrman EK , Kautzman AG , et al. Microglia sculpt postnatal neural circuits in an activity and complement‐dependent manner. Neuron. 2012;74(4):691‐705. PubMed PMID: 22632727. Pubmed Central PMCID: 3528177. 22632727 10.1016/j.neuron.2012.03.026 PMC3528177

[68] Bialas AR , Stevens B . TGF‐beta signaling regulates neuronal C1q expression and developmental synaptic refinement. Nat Neurosci. 2013;16(12):1773‐1782. PubMed PMID: 24162655. Pubmed Central PMCID: 3973738. 24162655 10.1038/nn.3560 PMC3973738

[69] Shatz CJ . MHC class I: an unexpected role in neuronal plasticity. Neuron. 2009;64(1):40‐45. PubMed PMID: 19840547. Pubmed Central PMCID: 2773547. 19840547 10.1016/j.neuron.2009.09.044 PMC2773547

[70] Lee H , Brott BK , Kirkby LA , et al. Synapse elimination and learning rules co‐regulated by MHC class I H2‐Db. Nature. 2014;509(7499):195‐200. PubMed PMID: 24695230. Pubmed Central PMCID: 4016165. 24695230 10.1038/nature13154 PMC4016165

[71] Head SR , Komori HK , Hart GT , et al. Method for improved Illumina sequencing library preparation using NuGEN ovation RNA‐Seq system. Biotechniques. 2011;50(3):177‐180. PubMed PMID: 21486238. 21486238 10.2144/000113613

[72] Zhou Y , Zhou B , Pache L , et al. Metascape provides a biologist‐oriented resource for the analysis of systems‐level datasets. Nat Commun. 2019;10(1). 10.1038/s41467-019-09234-6. PMC6447622 30944313

[73] Zhou Y , Zhou B , Pache L , et al. Metascape provides a biologist‐oriented resource for the analysis of systems‐level datasets. Nat Commun. 2019;10(1):1523 PubMed PMID: 30944313. Pubmed Central PMCID: 6447622. 30944313 10.1038/s41467-019-09234-6 PMC6447622

[74] Jassal B , Matthews L , Viteri G , et al. The reactome pathway knowledgebase. Nucleic Acids Res. 2020;48(D1):D498‐D503. PubMed PMID: 31691815. Pubmed Central PMCID: 7145712. 31691815 10.1093/nar/gkz1031 PMC7145712

[75] Shi Z , Piccus Z , Zhang X , et al. miR‐9 regulates basal ganglia‐dependent developmental vocal learning and adult vocal performance in songbirds. eLife. 2018;7:e29087. PubMed PMID: 29345619. Pubmed Central PMCID: 5800847. 29345619 10.7554/eLife.29087 PMC5800847

[76] Livak Kenneth J , Schmittgen Thomas D . Analysis of relative gene expression data using real‐time quantitative PCR and the 2−ΔΔCT method. Methods. 2001;25(4):402‐408. 10.1006/meth.2001.1262. 11846609

