# Supplementary figures and images for: Dynamic transcriptome landscape in the song nucleus HVC between juvenile and adult zebra finches
Source: Adv Genet (Hoboken). 2021 Jan 6;2(1):e10035. doi: 10.1002/ggn2.10035 (PMC9744550; doi:10.1002/ggn2.10035)

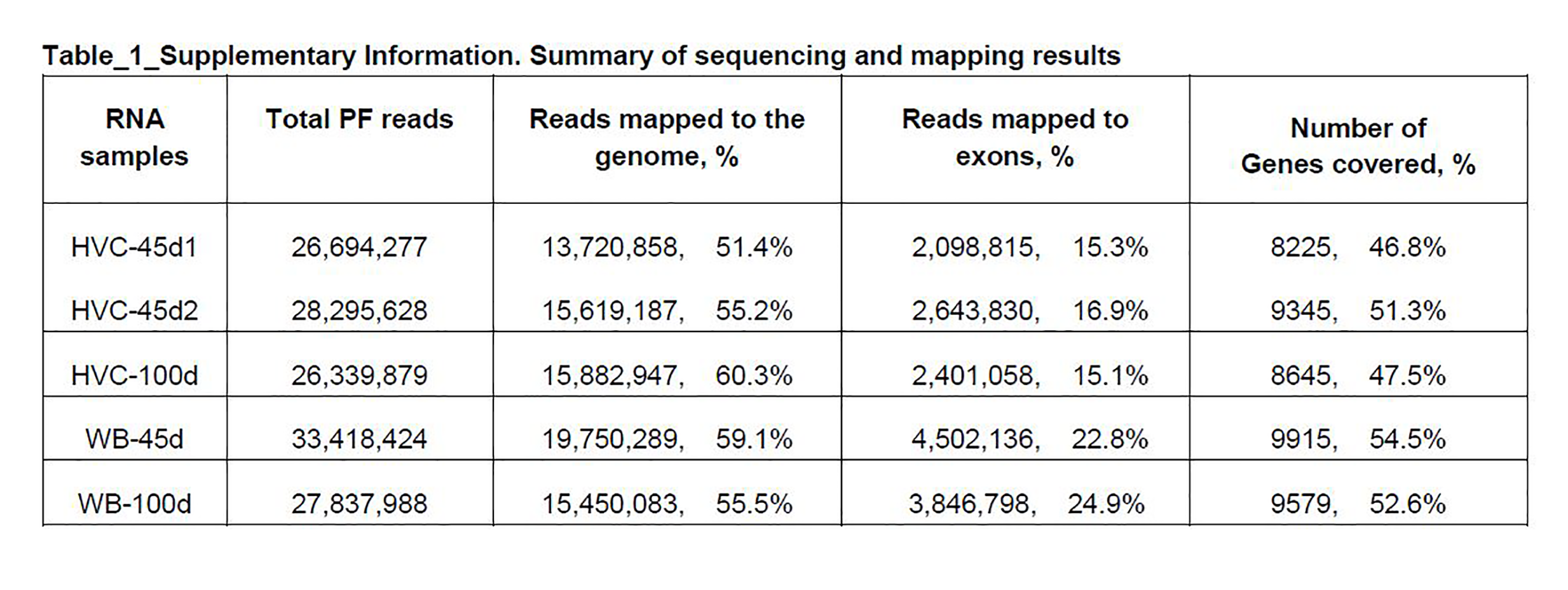

Supplement: Supplementary file 1 — Table S1 Summary of sequencing and mapping results [file GGN2-2-e10035-s005.tif]
